# One year after ICU admission for severe community-acquired pneumonia of bacterial, viral or unidentified etiology. What are the outcomes?

**DOI:** 10.1371/journal.pone.0243762

**Published:** 2020-12-14

**Authors:** Frédéric Sangla, David Legouis, Pierre-Emmanuel Marti, Sebastian D. Sgardello, Amélie Brebion, Pierre Saint-Sardos, Mireille Adda, Alexandre Lautrette, Bruno Pereira, Bertrand Souweine

**Affiliations:** 1 Service de Médecine intensive et réanimation, Centre Hospitalier Universitaire Gabriel Montpied, Clermont-Ferrand, France; 2 Service de Soins intensifs adultes, Hôpitaux Universitaires de Genève, Geneva, Switzerland; 3 Laboratoire de Virologie, Centre Hospitalier Universitaire Gabriel Montpied, Clermont-Ferrand, France; 4 Laboratoire de Bactériologie, Centre Hospitalier Universitaire Gabriel Montpied, Clermont-Ferrand, France; 5 Département de Biostatistique, Centre Hospitalier Universitaire Gabriel Montpied, Clermont-Ferrand, France; University of Notre Dame Australia, AUSTRALIA

## Abstract

**Introduction:**

Multiplex polymerase chain reaction (mPCR) for respiratory virus testing is increasingly used in community-acquired pneumonia (CAP), however data on one-year outcome in intensive care unit (ICU) patients with reference to the causative pathogen are scarce.

**Materials and methods:**

We performed a single-center retrospective study in 123 ICU patients who had undergone respiratory virus testing for CAP by mPCR and with known one-year survival status. Functional status including dyspnea (mMRC score), autonomy (ADL Katz score) and need for new home-care ventilatory support was assessed at a one-year post-ICU follow-up. Mortality rates and functional status were compared in patients with CAP of a bacterial, viral or unidentified etiology one year after ICU admission.

**Results:**

The bacterial, viral and unidentified groups included 19 (15.4%), 37 (30.1%), and 67 (54.5%) patients, respectively. In multivariate analysis, one-year mortality in the bacterial group was higher compared to the viral group (HR 2.92, 95% CI 1.71–7.28, *p* = 0.02) and tended to be higher compared to the unidentified etiology group (*p* = 0.06); but no difference was found between the viral and the unidentified etiology group (*p* = 0.43). In 64/83 one-year survivors with a post-ICU follow-up consultation, there were no differences in mMRC score, ADL Katz score and new home-care ventilatory support between the groups (*p* = 0.52, *p* = 0.37, *p* = 0.24, respectively). Severe dyspnea (mMRC score = 4 or death), severe autonomy deficiencies (ADL Katz score ≤ 2 or death), and major adverse respiratory events (new home-care ventilatory support or death) were observed in 52/104 (50.0%), 47/104 (45.2%), and 65/104 (62.5%) patients, respectively; with no difference between the bacterial, viral and unidentified group: *p* = 0.58, *p* = 0.06, *p* = 0.61, respectively.

**Conclusions:**

CAP of bacterial origin had a poorer outcome than CAP of viral or unidentified origin. At one-year, impairment of functional status was frequently observed, with no difference according to the etiology.

## Introduction

Severe community-acquired pneumonia (CAP) usually requires intensive care unit (ICU) admission, mainly because of septic shock or the need for ventilatory support [1]. Severe CAP is frequent [2, 3] and of increasing incidence over the last decades [4]. It is associated with high short-term [5, 6] and long-term [7, 8] mortality rates and with other long-term poor outcomes such as alterations of respiratory function and reduced autonomy [9]. A good understanding of the prognostic factors of severe CAP therefore has a significant interest. Studies on the long-term outcomes of severe CAP have reported factors associated with long term-mortality and autonomy. Among potential prognostic factors, the role of the causative pathogens in severe CAP is debated [10]. New diagnostic tools are now available to identify the etiological microorganisms of CAP, and in numerous cases the respiratory viruses are involved. The routine use of multiplex polymerase chain reaction (mPCR) has allowed the detection of respiratory viruses in 23% to 57% of severe CAP cases, as single etiologic agents or in combination with bacteria [10–14]. Questions remain about the effect of the causative microorganism on short-term mortality in patients with severe CAP [10, 12–14]. With regard to long-term outcomes, two studies performed in patients admitted to hospital medical departments for CAP reported no relationship between causative pathogens and mortality [15, 16]. Whether the results of these latter studies can be extrapolated to patients admitted to ICU for severe CAP remains unknown. PCR for respiratory virus testing has been available in our hospital since 2012 to diagnose viral pneumonia and is systematically used in patients admitted to the medical ICU with a suspicion of CAP. We therefore decided to compare one-year mortality and functional status in ICU patients admitted for bacterial CAP, viral CAP and CAP with no identified microorganisms.

## Materials and methods

### Patients

We performed a retrospective single-center cohort study in adult (>18 years) patients admitted to the 10-bed medical ICU of the University Hospital of Clermont-Ferrand (France) between 01 February 2013 and 31 July 2015 who had undergone mPCR for respiratory virus testing within 96 hours following ICU admission. The patients were screened from the databases of the local ICU and microbiological departments. Patients with an ICU length of stay > 24 hours, without aspiration pneumonia or pneumonia of fungal origin, and having a final diagnosis of CAP (including those diagnosed with Health Care-Associated Pneumonia (HCAP) were eligible and included if their survival status one year after ICU discharge was known. The final diagnosis of CAP was established by two external physicians (FS and PEM).

This study was approved by our institutional review board (Institutional review Board of Clermont-Ferrand South-East 6 –IRB 00008526, number 2019/CE24), which waived the need for written informed consent of the participants, in accordance with French legislation on noninterventional studies. However, the patients and their next of kin were informed about the inclusion of their anonymized medical data in the database and none asked for their withdrawal.

### Data collection

Data on ICU stay, hospital stay and post-ICU follow-up consultations performed one year after ICU admission were extracted from medical records (S1 Appendix). The data recorded one year after ICU admission included mortality, dyspnea assessed by the modified Medical Research Council (mMRC) dyspnea scale [17], new home-care ventilatory support and autonomy assessed by the Activities of Daily Living (ADL) Katz scale [18] (S2 Appendix). Data on long term-mortality were collected by telephone interviews of the patients’ family physicians.

### Microbiology

Respiratory viruses were tested by mPCR either on nasopharyngeal swabs or on lower respiratory tract specimens usually from bronchoalveolar lavage (BAL) fluid or endotracheal aspirates. Two different mPCR kits were used during the study period. For bacterial pathogens, respiratory tract samples underwent Gram staining and quantitative culture. Urine antigen testing was used to screen for *Streptococcus pneumoniae* and *Legionella pneumophila* and immunoglobulin antibody testing for *Chlamydia pneumoniae* and *Mycoplasma pneumoniae* (S3 Appendix).

### Definitions

Home-care ventilatory support was defined as either requirement for oxygen, continuous positive airway pressure (CPAP) or non-invasive ventilation (NIV) at home. New home-care ventilatory support was defined as requirement for home-care ventilatory support after hospital discharge in patients with no home-care ventilatory support before ICU admission, and need for CPAP or NIV after hospital discharge in patients having had oxygen at home before ICU admission. A major adverse respiratory event (MARE) was defined as the need for new home-care ventilatory support or all-cause death.

The viral group was defined as patients with one or more viruses identified as causative agents of CAP and with no bacteria identified, the bacterial group as patients with one or more bacteria identified as causative agents and with no viruses identified, and the unidentified etiology group as patients with neither viruses nor bacteria identified as causative agents. Patients with CAP due to bacterial-viral coinfection were not included in the main analysis (S1 Table). Other definitions including that of CAP are given in S4 Appendix.

### Endpoints

The primary endpoint was the comparison of one-year mortality after ICU admission in patients with bacterial severe CAP (bacterial group), viral severe CAP (viral group) and CAP with no causative microorganism identified (unidentified etiology group). Secondary endpoints were the functional status (presence of dyspnea, occurrence of MARE and autonomy) assessed one year after ICU admission and the survival during the follow-up period. We also assessed ICU, hospital and 28-day mortality after ICU admission, and ICU and hospital length of stay.

### Statistical analysis

The analysis was conducted to compare patients and outcomes in the three patient groups. All statistical analyses were performed with Stata software (version 13, StataCorp, College Station, US) for a two-sided type I error at 5%. Patients’ characteristics were expressed as number and percentage for categorical variables and as mean ± standard deviation or median (interquartile range) for quantitative variables, according to the statistical distribution (assumption of normality studied by the Shapiro-Wilk test). Comparisons between independent groups of continuous parameters were performed with ANOVA or Kruskal-Wallis tests if the assumptions of ANOVA were not met. Homoscedasticity was studied with the Bartlett test. Comparisons between groups concerning categorical variables were performed with chi-squared or Fisher's exact tests. For the ADL Katz and mMRC scores, deaths were included in the analysis and assigned to the worst value. A sensitivity analysis was performed in the subgroup of patients discharged alive from the hospital. Mortality analyses were performed with Cox proportional-hazards regression and the Kaplan-Meier method.

Multivariable analysis was performed with parameters associated with mortality in univariate analysis at a *p* value < 0.1 and with clinically relevant data such as a SOFA score ≥4 and chronic respiratory failure. The SOFA score was considered as a dichotomous variable categorized according to the median value. Particular attention was given to the study of the multicollinearity assessed using the Farrar-Glauber multicollinearity test and variance inflation factor. The proportional-hazard hypothesis was studied by Schoenfeld’s test. Plotting residuals and the interactions between possible prognostic factors were also tested. Hence, the results were expressed as hazard ratios (HR) and 95% confidence intervals.

## Results

**Fig 1:**
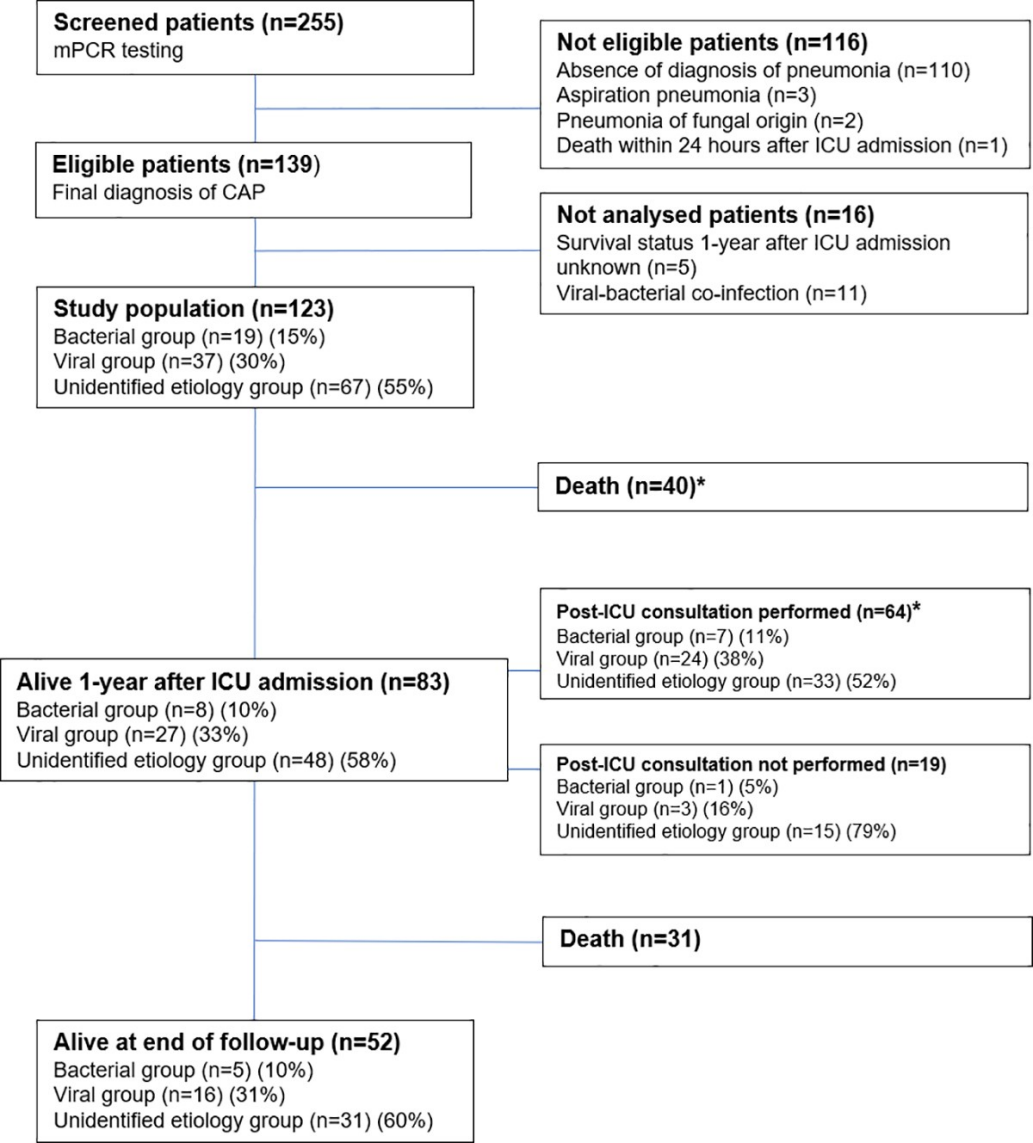
Flow chart. CAP = community-acquired pneumonia, ICU = Intensive care unit. *Patients included in the analysis of functional status. Deaths were assigned to the worst value for each functional score.

A total of 139 patients with a screened mPCR developed CAP (Fig 1). Five patients were excluded from the analysis because one-year survival status was not obtained. Eleven were excluded because they developed a mixed bacterial / viral CAP. Therefore, 123 patients with a known one-year survival status were considered for the main analysis. Among the 83 one-year survivors, 64 had a one-year follow-up consultation allowing for an evaluation of functional status, and in 19, vital status was the only post-hospital discharge information available. The characteristics of the 123 patients are shown in Table 1.

**Table 1:** Baseline characteristics and outcome of the 123 patients analyzed.

Patients	All patients (n = 123)	Bacterial group (n = 19)	Viral group (n = 37)	Unidentified etiology group (n = 67)	*p value* ^1^
Demographics and underlying diseases					
Age (years), median (IQR)	70 (63–80)	69 (60–75)	69 (63–75)	71 (66–81)	0.2
Sex, male, n (%)	75 (61)	13 (68.4)	18 (48.6)	44 (65.7)	0.2
Mc Cabe, median (IQR)	1 (1–2)	1 (1–2)	1 (1–2)	1 (1–1)	0.39
Immunocompromized patients, n (%)	25 (20.3)	4 (21.1)	8 (21.6)	13 (19.4)	0.95
HIV, n (%)	1 (0.8)	0 (0)	1 (2.7)	0 (0)	0.45
Daily corticosteroid therapy, n (%)	4 (3.3)	1 (5.3)	1 (2.7)	2 (3)	0.81
Other immunosuppressive therapy, n (%)	1 (0.8)	0 (0)	0 (0)	1 (1.5)	0.99
Solid organ transplantation, n (%)	2 (1.6)	0 (0)	1 (2.7)	1 (1.5)	0.99
Active cancer, n (%)	9 (7.3)	1 (5.3)	4 (10.8)	4 (6)	0.63
Malignant hemopathy, n (%)	8 (6.5)	2 (10.5)	1 (2.7)	5 (7.5)	0.42
Active smoker, n (%)	36 (29.3)	7 (36.8)	15 (40.5)	14 (20.9)	0.08
Diabetes Mellitus, n (%)	34 (27.6)	4 (21)	13 (35.1)	17 (25.4)	0.47
COPD, n (%)	43 (35)	5 (26.3)	19 (51.3)	19 (28.4)	0.06
Asthma, n (%)	8 (6.5)	0 (0)	2 (5.4)	6 (9)	0.55
Chronic respiratory failure ^2^, n (%)	28 (22.8)	4 (21.5)	15 (40.5)	9 (13.4)	0.008
Ventilatory support at home, n (%)	21 (17.1)	5 (26.3)	6 (16.2)	10 (14.9)	0.54
Chronic heart failure ^3^, n (%)	30 (23.4)	4 (21)	11 (29.7)	15 (22.4)	0.71
Antibiotics before ICU admission, n (%)	66 (53.7)	8 (42.1)	20 (54)	38 (56.7)	0.54
Characteristics at ICU admission					
Glasgow coma scale, median (IQR)	15 (14–15)	14 (5–15)	15 (15–15)	15 (14–15)	0.052
Temperature (°c), median (IQR)	37.3 (36.8–38)	37.2 (36.5–37.7)	37.2 (37.1–37.5)	37.3 (36.8–38)	0.45
Respiratory rate (breath/min), median (IQR)	24 (20–30)	28 (20–34)	22 (19–25)	25 (21–31)	0.07
Invasive mechanical ventilation, n (%)	14 (11.4)	5 (26.3)	3 (8.1)	6 (9)	0.13
PaO2/FiO2 (mmHg), median (IQR)	147 (93–236)	92 (85–177)	210 (125–276)	147 (93–230)	0.01
Lactate (mmol/L), median (IQR)	1.4 (1–2)	1.8 (1–2.5)	1.3 (0.8–1.8)	1.4 (1.1–2)	0.12
CRP (mg/L), median (IQR)	123 (66–219)	133 (66–320)	121 (57–191)	137 (72–205)	0.67
Procalcitonin (μg/L), median (IQR)	0.78 (0.28–3.4)	6.7 (0.51–12)	0.52 (0.18–1.3)	0.88 (0.29–3.25)	0.008
White blood cells (G/L), median (IQR)	10.6 (7.4–14.4)	10.1 (5.9–16)	9 (6.8–11)	11.7 (8.4–15.3)	0.027
Neutrophils (G/L), median (IQR)	8 (6–12.5)	10 (3.8–14.2)	8 (4.5–9)	9 (6–13.5)	0.47
Lymphocytes (G/L), median (IQR)	0.7 (0.4–1.3)	0.4 (0.3–1.3)	0.9 (0.5–1.3)	0.7 (0.5–1.2)	0.08
PSI score, median (IQR)	121 (89–142)	131 (91–156)	116 (84–145)	118 (89–141)	0.47
SAPS II score, median (IQR)	45 (38–62)	64 (39–80)	44 (36–51)	47 (38–62)	0.1
SOFA score, median (IQR)	4 (2–7)	6 (4–11)	3 (2–6)	4 (2–6)	0.013
Vasopressors, n (%)	38 (30.9)	11 (57.9)	12 (32.4)	25 (37.3)	0.16
HCAP, n (%)	20 (16.3)	1 (5.3)	7 (18.9)	12 (17.9)	0.44
During hospital stay					
Invasive mechanical ventilation, n (%)	39 (31.7)	11 (57.9)	10 (27)	18 (26.9)	0.046
ARDS, n (%)	17 (13.8)	7 (36.8)	3 (8.1)	7 (10.4)	0.01
Length of invasive mechanical ventilation (d), median (IQR)	6 (4–13)	5 (3–7)	10 (5–17)	8 (4–16)	0.33
Vasopressors, n (%)	103 (83.7)	13 (68.4)	33 (89.2)	57 (85.1)	0.1
Renal replacement therapy, n (%)	13 (10.6)	2 (10.5)	4 (10.8)	7 (10.5)	1
**Outcomes**					
ICU stay (d), median (IQR)	6 (4–11)	7 (3–11)	7 (5–13)	6 (4–10)	0.44
Hospital stay (d), median (IQR)	15 (10–23)	11 (6–21)	17 (10–22)	16 (10–23)	0.29
ICU mortality, n (%)	15 (12.2)	6 (31.6)	5 (13.5)	4 (6)	0.01
Day 28 mortality, n (%)	21 (17)	8 (42.1)	6 (16.2)	7 (10.5)	0.009
Hospital mortality, n (%)	27 (22)	8 (42.1)	7 (18.9)	12 (17.9)	0.07
One-year mortality, n (%)	40 (32.5)	11 (57.9)	10 (27.0)	19 (28.4)	0.046

Data are presented as mean (percentage) or as median (interquartile range).

ARDS = Acute respiratory distress syndrome, COPD = Chronic obstructive pulmonary disease, HCAP = Health care-associated pneumonia, HIV = Human immunodeficiency virus, ICU = Intensive care unit, IQR = Interquartile range, PSI = Pneumonia severity index, SAPSII = Simplified acute physiological score, SOFA = Sepsis-related organ failure assessment.

^1^ between the 3 groups.

^2^ Chronic respiratory failure was defined as the requirement of long-term oxygen therapy or chronic hypoxemia (defined as chronic arterial oxygen pressure lower than 70mmHg).

^3^ Chronic heart failure was defined as a physician’s diagnosis in the medical record.

**Fig 2:**
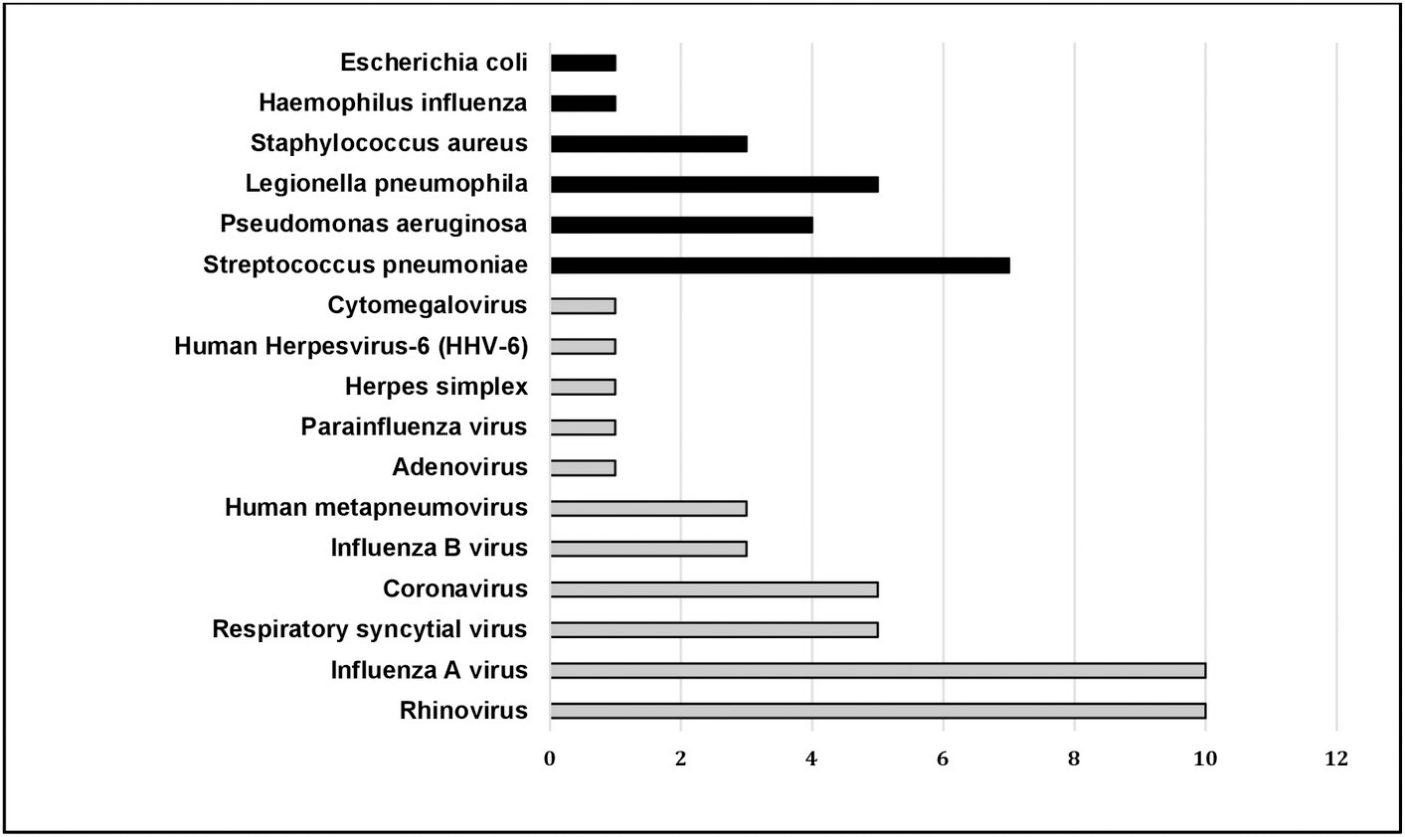
Microbiological findings in the 123 patients analyzed. Data are presented as number.

Microbiological investigations are described in the S2 Table. Bacterial blood cultures were performed on all but one patient. Bacterial culture of respiratory sample was performed in bacterial, viral and unidentified etiology groups in respectively 84.2%, 54.1% and 56.7% (*p* = 0.06) (S2 Table). The distribution of identified microorganisms is described in Fig 2. A bacterial documentation was obtained in 8/66 (12.1%) patients who had received antibiotics before ICU admission and in 11/57 (19.3%) patients who had not (*p* = 0.34). In the bacterial group, 8 patients (42.1%) had received antibiotics before ICU admission and 11 (57.9%) had not; blood cultures were performed in all 19 patients and were found positive in 4 (S2 Table). In the five patients on home ventilatory support, the identified microorganisms were *Pseudomonas aeruginosa* (n = 3), Methicillin sensitive *Staphylococcus aureus* (n = 1), and *Streptococcus pneumoniae* (n = 1).

The cumulative one-year mortality was 32.5% (Table 1). In univariate analysis, the factors significantly associated with one-year mortality were bacterial group (*p* = 0.004), immunocompromised patients (*p* = 0.007), chronic heart failure (*p* = 0.007) and higher SOFA score (*p*<0.001) (S3 Table). In the bacterial group there was no difference in one-year mortality between patients with and without bacteremia: (3/4) vs (8/15) respectively, (*p* = 0.603).

**Fig 3:**
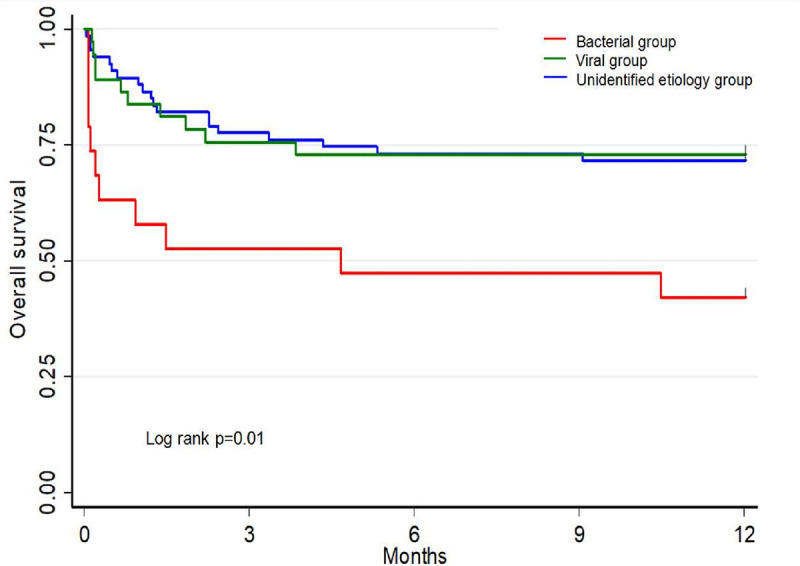
Kaplan-Meier plots of one-year survival after ICU admission in the three groups.

Fig 3 shows the Kaplan-Meier curve of one-year survival.

**Fig 4:**
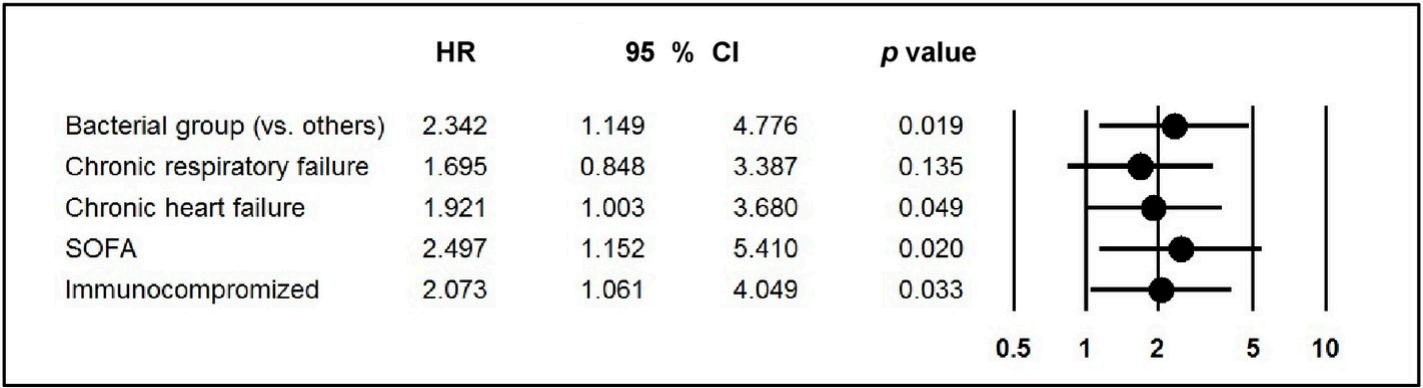
Multivariate analysis of factors associated with one-year mortality of 123 patients analyzed. HR = Hazard ratio, SOFA = Sepsis-related organ failure assessment, 95% CI 95% confidence interval.

In multivariate analysis, after adjustment for factors identified in the univariate analysis (immunocompromised patients, chronic heart failure and SOFA score) and for chronic respiratory failure, the bacterial group was still associated with higher one-year mortality (HR 2.34, 95%CI 1.15–4.78, *p* = 0.02) (Fig 4); similar results were observed when the variables age and diabetes were forced into the multivariate model (S1 Fig).

In univariate analysis of the three groups, one-year mortality was higher in the bacterial group than in the viral group (HR 2.86, 95%CI 1.21–6.74, *p* = 0.02) and the unidentified etiology group (HR 2.77, 95%CI 1.31–5.82, *p* = 0.007) with no differences in one-year mortality between the unidentified etiology and viral groups (HR 0.97, 95%CI 0.45–2.08, *p* = 0.93).

In multivariate analysis, one-year mortality was still higher in the bacterial group than in the viral group (HR 2.92, 95%CI 1.71–7.28, *p* = 0.02) whereas the difference was only marginal between the bacterial and unidentified etiology groups (HR 2.07, 95%CI 0.96–4.45, *p* = 0.06), and no difference was observed between the viral and unidentified etiology groups (HR 0. 71, 95%CI 0.30–1.65, *p* = 0.43).

Similar results were observed in a sensitivity analysis that restricted the initial viral group to patients with either a PCR positive to respiratory viruses performed on lower respiratory tract specimens or a PCR positive to RVS or influenza virus performed on nasal swabs (n = 20/37) and that reclassified the other patients of the initial viral group (n = 17/37) into the unidentified etiology group.

The median long-term follow-up in the 123 patients was 24.5 months (IQR 2.3–42.1). Long-term mortality was higher in the bacterial group than in the viral (HR 2.13, 95%CI 1.08–4.21, *p* = 0.03) and unidentified etiology groups (HR 2.17, 95%CI 1.17–4.04, *p* = 0.01). There were no differences in long-term mortality between the viral and unidentified etiology groups (HR 1.02, 95%CI 0.59–1.74, *p* = 0.95). A Kaplan-Meier curve of long-term survival is shown in S2 Fig.

One year after ICU admission, functional status was assessed in the 64 patients who had received a post-ICU follow-up consultation (Fig 1). The comparison of functional status in the groups when deaths (n = 40) were included and assigned to the worst value is given in Table 2. The results of the mMRC score, the MARE rate and the ADL Katz score were not different between groups (*p* = 0.58, *p* = 0.61 and *p* = 0.06, respectively) (Table 2).

**Table 2:** mMRC score, MARE and ADL Katz score one year after ICU admission in the 104 patients who had received post-ICU follow-up consultation or were dead.

Patients	All patients (n = 123)	Bacterial group (n = 19)	Viral group (n = 37)	Unidentified etiology group (n = 67)	*p* value
Analyzed population (n)	104	18	34	52	
Post-ICU consultation not performed (n)	19	1	3	15	
Post-ICU consultation performed (n)	64	7	24	33	
Death 1 year after ICU admission (n)	40	11	10	19	
mMRC score^a^^,^^b^, n (%)					0.58
score ≤1	29 (27.9)	4 (22.2)	9 (26.5)	16 (30.8)	
score = 2, n (%)	5 (4.8)	0	2 (5.9)	3 (5.8)	
score = 3, n (%)	18 (17.3)	3 (16.7)	9 (26.5)	6 (11.5)	
Score = 4, n (%)	52 (50)	11 (61.1)	14 (41.2)	27 (51.9)	
MARE^c^, n (%)	65 (62.5)	12 (66.7)	23 (67.7)	30 (57.7)	0.61
ADL Katz score ^b^^,^^d^, n (%)					0.06
Score = 6	35 (33.7)	5 (27.8)	16 (47.1)	14 (27)	
Score ≤5 and ≥3	22 (21.2)	1 (5.6)	6 (17.6)	15 (28.8)	
Score ≤2	47 (45.2)	12 (66.7)	12 (35.3)	23 (44.2)	

Data are presented as mean (percentage).

ADL = Activities of daily living, ICU = Intensive care unit, MARE = Major adverse respiratory event, mMRC = modified Medical research council.

^a^ The mMRC scale assesses dyspnea using a 5-point scale based on the sensation of breathing difficulty during daily life activities. Level 0 is the lowest level of perceived dyspnea and level 4 the greatest level of perceived dyspnea.

^b^ Dead patients were included and assigned to the worst value.

^c^ MARE was defined as the need for new home-care ventilatory support or as the all-cause death.

^d^ The ADL Katz scale assesses functional status as a measurement of the ability to perform activities of daily living (bathing, dressing, toileting, transferring, continence and feeding) independently. Patient score: Yes equals 1 point and No equals 0 point for independence in each of the six activities. A score of 6 indicates no deficiencies, 5 to 3 indicates mild to moderate deficiencies and 2 to 0 indicates severe deficiencies.

Of the 64 survivors who received a post-ICU follow-up consultation one year after ICU admission, 12 (18.8%) had severe dyspnea (mMRC score = 4), 25 (39%) had new home-care ventilatory support and 7 (10.9%) had severe autonomy deficiencies (ADL Katz score ≤ 2) (S4 Table).

New home-care ventilatory support was administered to 25 patients, of whom 20 had not had home-care ventilatory support before ICU admission (1 in the bacterial group, 12 in the viral group and 7 in the unidentified etiology group) and 5 who had (1 in the viral group and 4 in the unidentified etiology group). Among the 64 one-year survivors, there were no differences between groups in mMRC score, new home-care ventilatory support rates and the ADL Katz score (S4 Table).

## Discussion

The main findings of our study are as follows. First, patients with severe CAP in the bacterial group were more severely ill and had a higher one-year mortality than those in the viral group. Second, major dyspnea, MARE and deficiency of autonomy one year after admission were frequently observed in all three groups at comparable rates.

The bacterial group accounted for only 15.4% of our population while in most studies it accounts for 26% to 43% [10, 13, 14]. The low rate of bacterial CAP could reflect the lack of an extensive bacteriological diagnostic workup in several patients as suggested by a higher rate of respiratory bacterial cultures performed in this group (S2 Table), the exclusion of patients with bacterial and viral co-infection, and the large number of patients having received antibiotics before ICU admission (53.7%). In previous reports, the number of patients who had received antibiotics before admission was lower than in our study and ranged from 36% to 44% [10, 13, 14].

The unidentified etiology group accounted for 54.5% of our population while in most studies it ranges from 8% to 66% [10, 13, 14, 19, 20]. This could reflect differences in patient populations, definitions of severe CAP, diagnostic tools, timing of sampling and proportion of patients receiving antibiotics before sampling. A total of 56.7% of patients in the unidentified etiology group had received antibiotics before ICU admission and we cannot exclude that the negative bacterial test results observed in these patients corresponded to false-negative results.

Likewise, since the overall characteristics of patients in the unidentified etiology group are very close to those of the viral group, we cannot exclude that the absence of viral detection in the unidentified etiology group corresponded to false-negative viral PCR results. This assumption, however, remains speculative.

In our study, the viral group accounted for 30.1% of patients, which is consistent with the 23–43% rate of severe CAP of viral etiology published in other series [10, 11, 13, 14, 19]. As previously reported, influenza and rhinovirus were the leading causative viruses [11, 13, 19, 21]. We arbitrarily considered that all viruses identified on respiratory swabs were causative agents of CAP whatever their type and the site of sampling and therefore we cannot exclude that viral CAP was overdiagnosed. When influenza virus and respiratory syncytial virus (RSV) are identified in patients with signs of CAP by mPCR irrespective of the sampling site, they are classically considered as causative agents of CAP. In contrast, the pathogenic role of other respiratory viruses in CAP when they are not identified from a lower respiratory tract sample is debatable [22]. However, our sensitivity analysis showed no difference in one-year mortality between the viral and the unidentified etiology groups when the 17/37 patients with viruses different from RSV or influenza and not obtained from a distal sampling were reclassified into the unidentified etiology group (*p* = 0.72).

The 22% overall hospital mortality rate of severe CAP observed in our study is close to the rates classically reported, which range between 25% and 50% [23–25]. In our study, the mortality rates in the viral and unidentified etiology groups are consistent with those of previously published studies. However, the mortality rates in the bacterial group were higher than those previously reported [10, 13, 26]. This discrepancy could be related to the very low mortality rates of bacterial CAP reported in these works, which range between 10% and 25% [10, 13, 14, 26].

Previous reports found no differences of short-term mortality between patients with CAP of bacterial, viral or unidentified etiology [10, 13]. In contrast, we observed a higher mortality rate in the bacterial group. However, these patients were more severely ill than those in the other groups, as documented by higher SOFA scores, greater mechanical ventilation requirement and higher rates of ARDS.

Data on long-term outcome of severe CAP are scarce and no comparison has been made of the long-term outcomes in severe CAP depending on whether the etiology was bacterial, viral or unidentified. In our study, we observed a greater one-year mortality rate in the bacterial group. Our results differ from those of recent studies reporting no difference in long-term mortality between CAP due to different causative organisms. However, these studies involved non-severe CAP patients [15, 16].

On the other hand, the loss of significance of higher one-year mortality in the bacterial group compared to the unidentified etiology group seen after adjustment must be interpreted with caution. Our work cannot demonstrate the association between bacterial detection and one-year mortality. Nonetheless, this does not mean that the association does not exist. Indeed, considering the low number of patients diagnosed with bacterial pneumonia and the fact that the “true” rate of bacterial infection is unknown, particularly with the amount of pre-ICU antibiotic administration in this subgroup as well as the fallibility of culturing practices, it could be possible that the loss of significance is due to a lack of statistical power.

The long-term effects of acute respiratory failure on functional outcomes have been reported in ICU patients admitted for acute exacerbation of chronic obstructive pulmonary disease (COPD) [27, 28], ARDS [29–34] and in patients receiving prolonged mechanical ventilation [35], but no study has compared the functional status of patients with severe CAP of bacterial, viral or unidentified etiology.

Nevertheless, our findings are in line with those of studies reporting frequent and severe long-term autonomy deficiencies in severely ill critical care patients after ICU discharge [36].

Long-term dyspnea is associated with an increased risk of hospitalization in chronic obstructive pulmonary disease patients [27, 28], but has rarely been described in severe CAP patients except in a small cohort study performed in survivors of influenza A(H1N1)-associated ARDS, which showed less severe dyspnea than in our cohort [29]. The difference could be due to the older age and the higher rate of comorbidities in our patients.

We used MARE (a composite endpoint combining all-cause deaths and new home-care ventilatory support) because it provides major information on respiratory outcome in patients admitted for acute respiratory failure, is easily and objectively measured, subject to little confounding, captures a greater percentage of patients with a meaningful poor outcome, and obviates the limitation of competing risks between the two criteria.

The present study has several limitations. First, the study was a single-center study and included a low number of cases, thus limiting the generalization of the results. Nonetheless, the statistical power was greater than 80% [37–39] and the strength of the statistical model was confirmed using a bootstrap approach.

Second, it was a retrospective study. Complete diagnostic workup and bacteriological sampling were not performed in all patients, so we cannot exclude that some patients with severe CAP but no viral testing had not been identified. However, extensive bacterial sampling including blood culture, respiratory sampling culture and urine antigen testing was performed in most patients.

In addition, the decision to perform a post-ICU follow-up consultation was not based on a priori protocol but was left to the physician’s discretion, which partly contributed to the large number of patients lost to follow-up (19/83, 23%) after one year, the rate of which, however, is consistent with that of other studies (10 to 45%) reporting long-term outcomes after ICU discharge [31, 34].

Third, because of the study design, the bacteriological tests used, the sites of sampling, extensive prior use of antibiotics, as well as the absence of an extensive bacterial work-up in all patients, we cannot exclude the presence of false-negative and false-positive findings. The development and systematic use of broad panel mPCR, which can diagnose a large variety of bacterial and viral respiratory infections, could decrease the rates of CAP of unidentified etiology in the future. Fourth, we did not record the cause of death in the 13 hospital survivors who did not survive at one-year. Such data could help to define specific preventive strategies.

## Conclusions

In severe bacterial CAP, short-term and long-term mortality were greater than in severe viral CAP and CAP of unidentified etiology. Impaired functional status is often observed at one year after ICU admission, and was not significantly different in the three groups. Further studies systematically using a complete microbiological diagnostic work-up with new molecular tools to explore both respiratory viruses and bacteria could help to better define the effects of the causative microorganisms on long-term outcomes in severe CAP patients.

## Supporting information

S1 Appendix Data collection. (PDF)

S2 Appendix Score descriptions. (PDF)

S3 Appendix Microbiology. (PDF)

S4 Appendix Other definitions. (PDF)

S1 Table Baseline characteristics and outcomes of the 134 patients including the 11 eligible patients with bacterial-viral coinfection. Data are presented as mean (percentage) or as median (interquartile range). ARDS = Acute respiratory distress syndrome, ASAT = aspartate aminotransferase, COPD = Chronic obstructive pulmonary disease, HCAP = Health care-associated pneumonia, HIV = Human immunodeficiency virus, ICU = Intensive care unit, IQR = Interquartile range, PSI = Pneumonia severity index, SAPSII = Simplified acute physiological score, SOFA = Sepsis-related organ failure assessment. ^1^ between the 4 groups. ^2^ Chronic respiratory failure was defined as the requirement of long-term oxygen therapy or chronic hypoxemia (defined as chronic arterial oxygen pressure lower than 70mmHg). ^3^ Chronic heart failure was defined as a physician’s diagnosis in the medical record.
(PDF)

S2 Table Microbiological investigations. Data are presented as mean (percentage) mPCR, multiplex Polymerase Chain Reaction; ^a^ In 19 patients, 21 etiologic agents were identified 26 times, by positive blood culture (n = 4), bronchoalveolar lavage (n = 1), bronchial aspirate (n = 2), distal protected sample (n = 4), sputum (n = 6), and by urine antigen testing (*Streptococcus pneumoniae* (n = 4); *Legionella pneumophila* (n = 5)). ^b^ Bacterial culture of respiratory sample is defined as a bacterial culture of bronchial aspirate or distal protected sample or bronchoalveolar lavage or sputum. ^c^ *Mycoplasma pneumoniae* was tested in 89/123 patients (72.4%), when combining patients who underwent either serologic tests or nasopharyngeal swab mPCR. ^d^ *Chlamydia pneumoniae* was tested in 90/123 patients (73.3%), when combining patients who underwent either serologic tests or nasopharyngeal swab mPCR.
(RTF)

S3 Table Univariate analysis of factors associated with one-year mortality of 123 patients analyzed. COPD = Chronic obstructive pulmonary disease, HCAP = Health care-associated pneumonia, HR = Hazard ratio, SOFA = Sepsis-related organ failure assessment, 95% CI 95% confidence interval.
(PDF)

S4 Table mMRC score, new home-care ventilator support, ADL Katz score in the 64 survivors who had received post-ICU follow-up consultation one-year after ICU admission. Data are presented as mean (percentage). ADL Activities of daily living, ICU Intensive care unit, mMRC, modified Medical research council. ^a^ The mMRC scale assesses dyspnea using a 5-point scale based on the sensation of breathing difficulty during daily life activities. Level 0 is the lowest level of perceived dyspnea and level 4 the greatest level of perceived dyspnea. ^b^ New home-care ventilatory support was defined as requirement of home-care ventilatory support after hospital discharge in patients with no home-care ventilatory support before ICU admission, and need for CPAP or NIV after hospital discharge in patients having oxygen at home before ICU admission. ^c^ The ADL Katz scale assesses functional status as a measurement of the ability to perform activities of daily living (bathing, dressing, toileting, transferring, continence and feeding) independently. Patient score: Yes equals 1 point and No equals 0 point for independence in each of the six activities. A score of 6 indicates no deficiencies, 5 to 3 indicates mild to moderate deficiencies and 2 to 0 indicates severe deficiencies.
(PDF)

S1 Fig Multivariate analysis of factors associated with one-year mortality of 123 patients analyzed when the variables age and diabetes were forced into the model. SOFA = Sepsis-related organ failure assessment.
(PDF)

S2 Fig Kaplan-Meier plots of long-term survival after ICU admission in the three groups. (PDF)
