# Supplementary material for: One year after ICU admission for severe community-acquired pneumonia of bacterial, viral or unidentified etiology. What are the outcomes?
Source: PLoS One. 2020 Dec 14;15(12):e0243762. doi: 10.1371/journal.pone.0243762 (PMC7735561; doi:10.1371/journal.pone.0243762)
Supplement: S1 Appendix — (PDF) [file pone.0243762.s001.pdf]

S1 Appendix: Data collection

All data were extracted from medical records, which contained demographic characteristics, comorbidities, ventilatory support at home (oxygen, continuous positive airway pressure [CPAP], non-invasive ventilation [NIV]), antibiotics administered before ICU admission, clinical features, biological findings, microbiological investigations, pneumonia severity index (PSI) score [1], simplified acute physiological score (SAPS) II [2], sequential organ failure assessment (SOFA) score [3], the need for vasopressor, invasive mechanical ventilation, renal replacement therapy, ICU and hospital length of stay, and ICU, hospital and 28-day mortality.

During their ICU stay, patients and their relatives were informed that they could be referred to post-ICU follow-up consultations. Data on outcomes after hospital discharge were extracted from records of post-ICU follow-up consultations performed one year after ICU admission. In our center, follow-up consultation is conducted at the discretion of the clinician in subgroups of ICU patients, including those with severe CAP. Data collected on functional status during follow-up consultations comprised dyspnea assessed by modified Medical Research Council dyspnea scale (mMRC) [4], new home-care ventilatory support and autonomy assessed by Activities of Daily Living ADL Katz scale (ADL) [5]. Data on long term-mortality were collected by telephone interviews of the patients' family physicians.

References

1. Fine MJ, Auble TE, Yealy DM et al. A prediction rule to identify low-risk patients with community-acquired pneumonia. *N Engl J Med*. 1997;336(4):243-50.

2. Le Gall JR, Lemeshow S, Saulnier F. A new Simplified Acute Physiology Score (SAPS II) based on a European/North American multicenter study. *JAMA*. 1993; 270(24):2957-63.

3. Vincent JL, Moreno R, Takala J, et al. The SOFA (Sepsis-related Organ FailureAssessment) score to describe organ dysfunction/failure. *Intensive Care Med*. 1996;22:707-10.9.

4. Fletcher CM, Elmes PC, Fairbairn AS et al.The Significance of Respiratory Symptoms and the Diagnosis of Chronic Bronchitis in a Working Population. *British Medical Journal*. 1959; 2(5147):257-66.

5. Katz S, Ford AB, Moskowitz RW, et al. Studies of illness in the aged. The index of ADL: a standardized measure of biological and psychosocial function. *JAMA*. 1963 Sep;185:914-19.
