# Supplementary material for: One year after ICU admission for severe community-acquired pneumonia of bacterial, viral or unidentified etiology. What are the outcomes?
Source: PLoS One. 2020 Dec 14;15(12):e0243762. doi: 10.1371/journal.pone.0243762 (PMC7735561; doi:10.1371/journal.pone.0243762)
Supplement: S2 Appendix — (PDF) [file pone.0243762.s002.pdf]

S2 Appendix: Score descriptions

Score modified Medical Research Council dyspnea scale (mMRC) [1]

The mMRC scale assesses dyspnea using a 5-point scale based on the sensation of breathing difficulty during daily life activities. Level 0 is the lowest level of perceived dyspnea and level 4 the greatest level of perceived dyspnea.

0	"I only get breathless with strenuous exercise."
1	"I get short of breath when hurrying on level ground or walking up a slight hill."
2	"On level ground, I walk slower than people of the same age because of breathlessness or have to stop for breath when walking at my own pace."
3	"I stop for breath after walking about 100 yards or after a few minutes on level ground."
4	"I am too breathless to leave the house, or I am breathless when dressing."

Score Activities of Daily Living ADL Katz scale (ADL) [2]

The ADL Katz scale assesses functional status as a measurement of the ability to perform activities of daily living (bathing, dressing, toileting, transferring, continence and feeding) independently. Patient score: Yes equals 1 point and No 0 point for independence in each of the six activities. A score of 6 indicates no deficiencies, 5 to 3 indicates mild to moderate deficiencies and 2 to 0 indicates severe deficiencies.

Bathing
1 bathes self completely or needs help in bathing only a single part of the body such as the back, genital area or disabled extremity.
0 needs help with bathing more than one part of the body, getting in or out of the tub or shower. Requires total bathing.

Dressing
1 gets clothes from closets and drawers and puts on clothes and outer garments complete with fasteners. May have help tying shoes.
0 needs help with dressing self or needs to be completely dressed.

Toileting
1 goes to toilet, gets on and off, arranges clothes, cleans genital area without help.
0 needs help transferring to the toilet, cleaning self or uses bedpan or commode.

Transferring
1 moves in and out of bed or chair unassisted. Mechanical transferring aides are acceptable.
0 needs help in moving from bed to chair or requires a complete transfer.

Continence
1 exercises complete self control over urination and defecation.
0 is partially or totally incontinent of bowel or bladder.

Feeding
1 gets food from plate into mouth without help. Preparation of food may be done by another person.
0 needs partial or total help with feeding or requires parenteral feeding.

References

1. Fletcher CM, Elmes PC, Fairbairn AS et al.The Significance of Respiratory Symptoms and the Diagnosis of Chronic Bronchitis in a Working Population. *British Medical Journal.* 1959; 2(5147):257-66.

2. Katz S, Ford AB, Moskowitz RW, et al. Studies of illness in the aged. The index of ADL: a standardized measure of biological and psychosocial function. *JAMA*. 1963 Sep;185:914-19.
