# Supplementary material for: One year after ICU admission for severe community-acquired pneumonia of bacterial, viral or unidentified etiology. What are the outcomes?
Source: PLoS One. 2020 Dec 14;15(12):e0243762. doi: 10.1371/journal.pone.0243762 (PMC7735561; doi:10.1371/journal.pone.0243762)
Supplement: S3 Appendix — (PDF) [file pone.0243762.s003.pdf]

S3 Appendix: Microbiology

Respiratory viruses were tested by polymerase chain reaction (PCR) either on nasopharyngeal swabs or on lower respiratory tract specimens, usually bronchoalveolar lavage fluid (BAL) or endotracheal aspirate. Detection was systematically performed of influenza A and B viruses, adenovirus (AdV), human parainfluenza virus (HPIV) (types 1, 2, 3 and 4), respiratory syncytial virus (RSV) types (A and B), picornavirus (rhinovirus and enterovirus), human metapneumovirus (HMPV) and human coronavirus (HCoV) types 229E, NL63, OC43 and HK1. Detection of bocavirus (HBoV) was performed from February 2013 to March 2014. Different PCR kits were used during the study period, the duplex Respiratory MWS R-GENE (Biomérieux, Marcy l'Etoile, France) PCR kits (influenza A/B, RSV/HMPV, rhinovirus and enterovirus, AdV/HBoV, HCoV/HPIV) from February 2013 to March 2014, and the Filmarray Respiratory Panel (BioFire Diagnostics®, Salt Lake City, USA), from April 2014 to August 2015. Of note, the Filmarray Respiratory panel comprises three bacterial targets, *Mycoplasma pneumoniae*, *Chlamydia pneumoniae* and *Bordetella pertussis*, but is unable to detect HBoV.

All picornavirus isolates were prospectively genotyped on the basis of phylogenetic analysis of the 1A/1B genomic region (A). All AdV isolates were prospectively genotyped on the basis of phylogenetic analysis of a partial region of the hexon genomic region (B). Lower respiratory tract specimens were screened systematically for herpes simplex viruses 1 and 2 (HSV-1, HSV-2) and cytomegalovirus (CMV) and for varicella zoster virus (VZV), Epstein Barr virus (EBV) and human herpesvirus 6 (HHV-6) at the physician's request. The HSV and VZV PCR used the HSV1 HSV2 VZV R-gene® kit (Argene, Verniolle, France) from February 2013 to October 2014 and the alpha Herpesviridae® kit (altona Diagnostics, Hamburg, Germany) from November 2014 to August 2015. The HHV-6 PCR used the CMV HHV-6,7,8 R-gene® kit (Argene, Verniolle, France) from February 2013 to October 2014

and the RealStar HHV-6 PCR® kit (altona Diagnostics, Hamburg, Germany) from November 2014 to August 2015. The CMV PCR used the CMV R-gene® kit (Argene, Verniolle, France) and the EBV PCR the EBV R-gene® kit (Argene, Verniolle, France). PCRs were performed according to the manufacturer's recommendations.

Respiratory tract samples underwent Gram staining and quantitative culture. Blood culture was performed in BD BACTEC™ Plus Aerobic and Anaerobic media & BD BACTEC™ Peds Plus™ medium with a Bactec 9000 system (Becton, Dickinson, USA). BinaxNOW kits (Alere, Jouy-en-Josas, France) were used for urine antigen testing of *Streptococcus pneumoniae* and *Legionella pneumophila*. The presence of *M. pneumoniae*- and *C. pneumoniae*-specific antibodies in serum samples was determined by ELISA with Platelia™ *M. pneumoniae* IgM & IgG kits (Bio-Rad, Marnes-la-Coquette, France) and *C. pneumoniae* IgG and IgM kits (Euroimmun, Luebeck, Germany).

For *C. pneumoniae* and *M. pneumoniae*, immunoglobulin antibody testing was considered positive if IgM antibodies were identified. The bacterium was considered as a causative pathogen of CAP if it fulfilled at least one of the following criteria: identified in blood culture or pleural fluid, in sputum specimen at ≥ 10^7 cfu/mL (with leucocyte > 25/field and epithelial cells < 10/field), in bronchoalveolar lavage fluid or bronchial aspirate specimen at ≥ 10^4 cfu/mL, in protected distal specimen (plugged telescoping catheter) at ≥ 10^3 cfu/mL. *S. pneumoniae* or *L. pneumophila* was considered as a pathogen whatever the type of test and level of positivity. *C. pneumoniae* or *M. pneumoniae* was considered as a pathogen after identification by Ig antibody test.
