# Supplementary material for: One year after ICU admission for severe community-acquired pneumonia of bacterial, viral or unidentified etiology. What are the outcomes?
Source: PLoS One. 2020 Dec 14;15(12):e0243762. doi: 10.1371/journal.pone.0243762 (PMC7735561; doi:10.1371/journal.pone.0243762)
Supplement: S4 Appendix — (PDF) [file pone.0243762.s004.pdf]

S4 Appendix: Other definitions

Pneumonia was defined by the association of at least two signs of systemic inflammatory response, a new radiological infiltrate and the occurrence of at least two clinical signs of pneumonia (cough, sputum, chest pain, respiratory distress, crackles, fever and hypoxemia (arterial oxygen saturation <90% or oxygen requirements > 5L/minute or mechanical ventilation). Pneumonia was considered as CAP when it had developed before hospital admission and as HCAP when it developed in nursing-home residents, patients in long-term care facilities, patients receiving home- or hospital-based intravenous therapy and patients undergoing chronic dialysis [1].

COPD and asthma were defined as a physician's diagnosis in the medical record.

Since pneumonia and COPD exacerbation can present similar clinical symptoms, COPD exacerbation was identified by the absence of radiological infiltrate [2].

Immunocompromized patients were patients with at least one immunosuppression factors as defined by the presence of human immunodeficiency virus (HIV)-positive testing, solid organ transplantation, hematopoietic stem cell transplantation, chemotherapy or radiotherapy for cancer within 6 months before admission, daily corticosteroid therapy> 5 mg/day of prednisone or equivalent or other immunosuppressive therapy and acquired immune deficiency (neutropenia <0.5G/L, hematological malignancy, active cancer).

Chronic respiratory failure was defined as the requirement of long-term oxygen therapy or chronic hypoxemia (defined as chronic arterial oxygen pressure lower than 70mmHg).

Chronic heart failure was defined as a physician's diagnosis in the medical record. Chronic neurological disease was defined as a central nervous disease (including any seizure disorder like epilepsy) or as a neuromuscular disease (including Parkinson's disease or muscular dystrophy). Chronic kidney disease was defined as a history of chronic renal disease. Mellitus diabetes was defined as a physician's diagnosis in the medical record. Acute respiratory

distress syndrome (ARDS) was defined according to Berlin definition [3]. Active smoker and alcohol consumption were defined as self-reported smoking or alcohol consumption. Antibiotics before ICU admission concerned any administration of antibiotics before ICU admission, whatever the drug regimen.

References

1. Kollef MH, Morrow LE, Baughman RP, et al. Health Care-Associated Pneumonia (HCAP): A Critical Appraisal to Improve Identification, Management, and Outcomes--Proceedings of the HCAP Summit. *Clinical Infectious Diseases: An Official Publication of the Infectious Diseases Society of America.* 2008 Apr;46 Suppl 4:S296-334; quiz 335-338.

2. Finney LJ, Padmanaban V, Todd S, et al. Validity of the diagnosis of pneumonia in hospitalised patients with COPD. *ERJ Open Res.* 2019 Jun 24;5(2).

3. ARDS Definition Task Force, Ranieri VM, Rubenfeld GD, et al. Acute Respiratory Distress Syndrome: The Berlin Definition. *JAMA.* 2012 Jun;307(23):2526-33.
