# Supplementary material for: One year after ICU admission for severe community-acquired pneumonia of bacterial, viral or unidentified etiology. What are the outcomes?
Source: PLoS One. 2020 Dec 14;15(12):e0243762. doi: 10.1371/journal.pone.0243762 (PMC7735561; doi:10.1371/journal.pone.0243762)
Supplement: S1 Table — Data are presented as mean (percentage) or as median (interquartile range). ARDS = Acute respiratory distress syndrome, ASAT = aspartate aminotransferase, COPD = Chronic obstructive pulmonary disease, HCAP = Health care-associated pneumonia, HIV = Human immunodeficiency virus, ICU = Intensive care unit, IQR = Interquartile range, PSI = Pneumonia severity index, SAPSII = Simplified acute physiological score, SOFA = Sepsis-related organ failure assessment. 1 between the 4 groups. 2 Chronic respiratory failure was defined as the requirement of long-term oxygen therapy or chronic hypoxemia (defined as chronic arterial oxygen pressure lower than 70mmHg). 3 Chronic heart failure was defined as a physician’s diagnosis in the medical record. (PDF) [file pone.0243762.s005.pdf]

S1 Table: Baseline characteristics and outcomes of the 134 patients including the 11 eligible patients with bacterial-viral coinfection

Patients	Bacterial group (n = 19)	Viral group (n = 37)	Unidentified etiology group (n = 67)	Bacterial-viral group (n = 11)	*p* value[1]
Demographics and underlying diseases					
Age (years), median (IQR)	69 (60-75)	69 (63-75)	71 (66-81)	69 (61-75)	0.29
Sex, male, n (%)	13 (68)	18 (47)	44 (66)	7 (64)	0.33
Weight, kg, median (IQR)	80 (65-91)	76 (61-91)	75 (63-86)	70 (59-85)	0.76
Immunocompromized patients, n (%)	4 (21)	8 (22)	13 (19)	2 (18)	0.99
COPD, n (%)	5 (26)	19 (51)	19 (28)	4 (36)	0.10
Asthma, n (%)	0 (0)	2 (5)	6 (9)	0 (0)	0.62
Chronic respiratory failure[2], n (%)	4 (21)	15 (41)	9 (13)	3 (27)	0.02
Ventilatory support at home, n (%)	5 (26)	6 (16)	10 (15)	4 (36)	0.54
Chronic neurological disease, n (%)	2 (11)	2 (5)	4 (6)	2 (18)	0.37
Chronic heart failure[3], n (%)	4 (21)	11 (30)	15 (22)	1 (9)	0.59
Chronic kidney disease, n (%)	1 (5)	8 (22)	7 (10)	3 (27)	0.13
Mellitus diabetes, n (%)	4 (21)	13 (35)	17 (25)	3 (27)	0.67
Cirrhosis, n (%)	2 (11)	3 (8)	3 (4)	0 (0)	0.55
Active smoker, n (%)	7 (37)	15 (41)	14 (21)	3 (27)	0.15
Alcohol consumption, n (%)	4(21)	5 (14)	7 (10)	1 (9)	0.61
Antibiotics before ICU admission, n (%)	8 (42)	20 (54)	38 (57)	5 (45)	0.67
Characteristics at ICU admission					
Glasgow coma scale, median (IQR)	14 (5-15)	15 (15-15)	15 (14-15)	15 (5-15)	0.01
PaO2/FiO2 (mmHg), median (IQR)	92 (85-177)	210 (125-276)	147 (93-230)	184 (137-286)	0.01
PSI score, median (IQR)	131 (91-156)	116 (84-145)	118 (89-141)	154 (106-159)	0.42
SOFA score, median (IQR)	6 (4-11)	3 (2-6)	4 (2-6)	5 (4-11)	0.02
SAPS II, median (IQR)	64 (39-80)	44 (36-51)	47 (38-62)	44 (33-73)	0.2
Temperature (°c), median (IQR)	37.2 (36.5-37.7)	37.2 (37.1-37.5)	37.3 (36.8-38)	37.2 (37.1-37.5)	0.63
Systolic blood pressure (mmHg), median (IQR)	107 (93-116)	123 (100-136)	118 (109-130)	124 (114-144)	0.057
Diastolic blood pressure (mmHg), median (IQR)	60 (59-76)	63 (54-69)	65 (57-77)	66 (59-86)	0.51
Mean blood pressure (mmHg), median (IQR)	70 (64-86)	76 (65-84)	77 (69-89)	81 (75-96)	0.31
Heart rate (beats/min), median (IQR)	108 (87-118)	90 (77-105)	97 (83-111)	99 (87-134)	0.22
Respiratory rate (b/min), median (IQR)	28 (20-34)	22 (19-25)	25 (21-31)	29 (18-35)	0.16
Diuresis (mL/h), median (IQR)	30 (16-115)	66 (38-107)	47 (25-112)	32 (24-90)	0.5
Bicarbonates (mmol/L), median (IQR)	22 (20-25)	27 (23-31)	25 (21-29)	23 (21-25)	0.15
Arterial pH, median (IQR)	7.35 (7.3-7.42)	7.35 (7.25-7.42)	7.4 (7.34-7.45)	7.37 (7.34-7.41)	0.09
Lactate (mmol/L), median (IQR)	1.8 (1-2.5)	1.3 (0.8-1.8)	1.4 (1.1-2)	2.2 (1.2-2.4)	0.17
Troponins (µg/L), median (IQR)	0.09 (0.04-0.13)	0.03 (0-0.14)	0.12 (0.03-1.12)	0.12 (0.02-1)	0.13
Urea (mmol/L) , median (IQR)	11.1 (5.6-14.4)	13 (6.4-22.7)	9.6 (6.3-19.8)	13.2 (11.4-21)	0.56
Creatinine (µmol/L), median (IQR)	105 (67-139)	106 (72-171)	94 (68-143)	134 (72-277)	0.74
Serum Na+ (mmol/L), median (IQR)	138 (132-140)	138 (134-140)	138 (134-141)	139 (137-142)	0.62
Serum K+ (mmol/L), median (IQR)	3.7 (3.3-4.1)	4.2 (3.8-4.6)	3.9 (3.6-4.3)	4.2 (3.8-4.9)	0.048
Glycemia (mmol/L), median (IQR)	6.8 (5.3-8.9)	7.5 (5.8-9)	6.6 (5.5-7.6)	8.5 (6.7-10.4)	0.22
CRP (mg/L), median (IQR)	133 (66-320)	121 (57-191)	137 (72-2015)	191 (115-234)	0.44
Procalcitonin (µg/L), median (IQR)	6.7 (0.51-12)	0.52 (0.18-1.3)	0.88 (0.29-3.3)	2.7 (0.76-4.5)	0.007
Lactate dehydrogenase, (U/L), median (IQR)	330 (191-473)	263 (212-335)	288 (236-430)	276 (225-389)	0.87
ASAT (U/L), median (IQR)	62 (26-112)	30 (21-47)	31 (19-67)	28 (22-137)	0.45

Bilirubin total (μmol/L), median (IQR)	12 (10-33)	8 (5-11)	8.5 (6-11)	11 (8-14)	0.02
White blood cells (G/L), median (IQR)	10.1 (5.9-16)	9 (6.8-11)	11.7 (8.4-15.3)	8.7 (6.5-11.6)	0.04
Neutrophils (G/L), median (IQR)	10 (3.8-14.2)	8 (5-9)	9 (6-14)	7 (5.2-11)	0.63
Lymphocytes (G/L), median (IQR)	0.4 (0.3-1.3)	0.9 (0.5-1.3)	0.7 (0.5-1.3)	0.35 (0.29-0.46)	0.02
Hematocrite (%), median (IQR)	36 (31-39)	35 (32-44)	34 (30-38)	33 (31-42)	0.31
Platelets (G/L), median (IQR)	169 (142-244)	185 (157-247)	219 (169-293)	199 (134-231)	0.11
Creatine kinase (U/L), median (IQR)	172 (91-581)	149 (57-357)	114 (64-210)	328 (54-383)	0.34
Invasive mechanical ventilation, n (%)	11 (58)	10 (27)	18 (27)	5 (45)	0.049
HCAP, n (%)	1 (5)	7 (19)	12 (18)	4 (36)	0.2
During hospital stay					
ARDS, n (%)	7 (37)	3 (8.)	7 (10)	0 (0)	0.01
Renal replacement therapy, n (%)	3 (16)	7 (19)	11 (16)	3 (27)	0.79
ICU stay (d), median (IQR)	7 (3-11)	7 (5-13)	6 (4-10)	11 (6-15)	0.11
Hospital stay (d), median (IQR)	11 (6-21)	17 (10-22)	16 (10-23)	16 (9-27)	0.43
ICU mortality, n (%)	6 (31.6)	5 (13.5)	4 (6)	1 (9.1)	0.03
Hospital mortality, n (%)	8 (42.1)	7 (18.9)	12 (17.9)	1 (9.1)	0.12
Day 28 mortality, n (%)	8 (42.1)	6 (16.2)	7 (10.5)	1 (9.1)	0.02

Data are presented as mean (percentage) or as median (interquartile range)

ARDS = Acute respiratory distress syndrome, ASAT = aspartate aminotransferase, COPD = Chronic obstructive pulmonary disease, HCAP = Health care-associated pneumonia, HIV = Human immunodeficiency virus, ICU = Intensive care unit, IQR = Interquartile range, PSI = Pneumonia severity index, SAPSII = Simplified acute physiological score, SOFA = Sepsis-related organ failure assessment

[1] between the 4 groups

[2] Chronic respiratory failure was defined as the requirement of long-term oxygen therapy or chronic hypoxemia (defined as chronic arterial oxygen pressure lower than 70mmHg)

[3] Chronic heart failure was defined as a physician’s diagnosis in the medical record
