# Supplementary material for: One year after ICU admission for severe community-acquired pneumonia of bacterial, viral or unidentified etiology. What are the outcomes?
Source: PLoS One. 2020 Dec 14;15(12):e0243762. doi: 10.1371/journal.pone.0243762 (PMC7735561; doi:10.1371/journal.pone.0243762)
Supplement: S2 Table — Data are presented as mean (percentage) mPCR, multiplex Polymerase Chain Reaction; a In 19 patients, 21 etiologic agents were identified 26 times, by positive blood culture (n = 4), bronchoalveolar lavage (n = 1), bronchial aspirate (n = 2), distal protected sample (n = 4), sputum (n = 6), and by urine antigen testing (Streptococcus pneumoniae (n = 4); Legionella pneumophila (n = 5)). b Bacterial culture of respiratory sample is defined as a bacterial culture of bronchial aspirate or distal protected sample or bronchoalveolar lavage or sputum. c Mycoplasma pneumoniae was tested in 89/123 patients (72.4%), when combining patients who underwent either serologic tests or nasopharyngeal swab mPCR. d Chlamydia pneumoniae was tested in 90/123 patients (73.3%), when combining patients who underwent either serologic tests or nasopharyngeal swab mPCR. (RTF) [file pone.0243762.s006.rtf]

S2 Table: Microbiological investigations 

Patients	All 	Bacterial 	Viral 	Unidentified 	p value	
	patients 	group 	group 	etiology group		
	(n = 123)	(n = 19)a	(n = 37)	(n = 67)		
mPCR total, n (%)	123 (100)	19 (100)	37 (100)	67 (100)	1	
mPCR in nasopharyngeal swabs, n (%)	117 (95.1)	17 (89.5)	36 (97.3)	64 (95.5)	0.36	
mPCR in bronchoalveolar lavage, n (%)	13 (10.6)	2 (10.5)	5 (13.5)	6 (9)	0.78	
Bacterial blood culture, n (%)	122 (99.2)	19 (100)	37 (100)	66 (98.5)	1	
Bacterial culture of respiratory sampleb, n (%)	74 (60.1)	16 (84.2)	20 (54.1)	38 (56.7)	0.06	
Bacterial culture of bronchial aspirate, n (%)	38 (30.9)	6 (31.6)	9 (24.3)	23 (34.3)	0.64	
Bacterial culture of distal protected sample, n (%)	32 (26.1)	9(47.4)	7 (18.9)	16 (23.9)	0.08	
Bacterial culture of bronchoalveolar lavage, n (%)	20 (16.3)	2 (10.5)	6 (16.2)	12 (17.9)	0.84	
Bacterial culture of sputum, n (%)	28 (22.8)	7 (36.8)	7 (18.9)	14 (20.9)	0.31	
Bacterial culture of pleural fluid, n (%)  	2 (1.6)	0 (0)	0 (0)	2 (3)	0.67	
Streptococcus pneumoniae urine antigen testing, n (%)	92 (74.8)	17(89.5)	27 (73)	48 (71.6)	0.30	
Legionella pneumophila urine antigen testing, n (%)	92 (74.8)	17 (89.5)	27 (73)	48 (71.6)	0.30	
Mycoplasma pneumoniaec serology, n (%)	46 (37.4)	8 (42.1)	10 (27)	28 (41.8)	0.31	
Chlamydia pneumoniaed serology, n (%)	49 (39.8)	8 (42.1)	12 (32.4)	29 (43.3)	0.54	

Data are presented as mean (percentage)
mPCR, multiplex Polymerase Chain Reaction
a In 19 patients, 21 etiologic agents were identified 26 times, by positive blood culture (n=4), bronchoalveolar lavage (n=1), bronchial aspirate (n=2), distal protected sample (n=4), sputum (n=6), and by urine antigen testing (Streptococcus pneumoniae (n=4); Legionella pneumophila, (n=5)).
b Bacterial culture of respiratory sample is defined as a bacterial culture of bronchial aspirate or distal protected bronchial sample or bronchoalveolar lavage or sputum.
c Mycoplasma pneumoniae was tested in 89/123 patients (72.4%), when combining patients who underwent either serologic tests or nasopharyngeal swab mPCR. 
d Chlamydia pneumoniae was tested in 90/123 patients (73.3%), when combining patients who underwent either serologic tests or nasopharyngeal swab mPCR.
