# Supplementary material for: One year after ICU admission for severe community-acquired pneumonia of bacterial, viral or unidentified etiology. What are the outcomes?
Source: PLoS One. 2020 Dec 14;15(12):e0243762. doi: 10.1371/journal.pone.0243762 (PMC7735561; doi:10.1371/journal.pone.0243762)
Supplement: S3 Table — COPD = Chronic obstructive pulmonary disease, HCAP = Health care-associated pneumonia, HR = Hazard ratio, SOFA = Sepsis-related organ failure assessment, 95% CI 95% confidence interval. (PDF) [file pone.0243762.s007.pdf]

S3 Table: Univariate analysis of factors associated with one-year mortality of 123 patients analyzed

Variables	HR (95% CI)	*p* value
Sex	1.56 (0.79-3.1)	0.196
Age	1.2 (0.1-1.05)	0.067
Weight	0.99 (0.97-1.002)	0.077
COPD	1.03 (0.54-1.97)	0.93
Chronic respiratory failure	1.63 (0.83-3.21)	0.157
Chronic heart failure	2.41 (1.23-4.55)	0.007
Chronic kidney disease	0.71 (0.25-1.99)	0.512
Chronic neurological disease	0.71 (1.17-2.93)	0.631
Cirrhosis	1.21 (0.37-3.9)	0.75
Alcohol consumption	0.77 (0.27-2.16)	0.62
Immunocompromized patients	2.44 (1.27-4.68)	0.007
HCAP	1.1 (0.49-2.48)	0.821
Antibiotics before ICU admission	1.01 (0.54-1.9)	0.99
SOFA score	1.16 (1.01-1.26)	<0.001

COPD = Chronic obstructive pulmonary disease, HCAP = Health care-associated pneumonia, HR = Hazard ratio, SOFA = Sepsis-related organ failure assessment, 95% CI 95% confidence interval
