# Supplementary material for: One year after ICU admission for severe community-acquired pneumonia of bacterial, viral or unidentified etiology. What are the outcomes?
Source: PLoS One. 2020 Dec 14;15(12):e0243762. doi: 10.1371/journal.pone.0243762 (PMC7735561; doi:10.1371/journal.pone.0243762)
Supplement: S4 Table — Data are presented as mean (percentage). ADL Activities of daily living, ICU Intensive care unit, mMRC, modified Medical research council. a The mMRC scale assesses dyspnea using a 5-point scale based on the sensation of breathing difficulty during daily life activities. Level 0 is the lowest level of perceived dyspnea and level 4 the greatest level of perceived dyspnea. b New home-care ventilatory support was defined as requirement of home-care ventilatory support after hospital discharge in patients with no home-care ventilatory support before ICU admission, and need for CPAP or NIV after hospital discharge in patients having oxygen at home before ICU admission. c The ADL Katz scale assesses functional status as a measurement of the ability to perform activities of daily living (bathing, dressing, toileting, transferring, continence and feeding) independently. Patient score: Yes equals 1 point and No equals 0 point for independence in each of the six activities. A score of 6 indicates no deficiencies, 5 to 3 indicates mild to moderate deficiencies and 2 to 0 indicates severe deficiencies. (PDF) [file pone.0243762.s008.pdf]

S4 Table: mMRC score, new home-care ventilator support, ADL Katz score in the 64 survivors who had received post-ICU follow-up consultation one-year after ICU admission

Patients	All patients (n = 83)	Bacterial group (n = 8)	Viral group (n = 27)	Unidentified etiology group (n = 48)	*p* value
Post-ICU consultation not performed (n)	19	1	3	15	
Post-ICU consultation performed (n)	64	7	24	33	
mMRC score [a], n (%)					0.52
score ≤1	29 (27.9)	4 (22.2)	9 (26.5)	16 (30.8)	
score =2	5 (4.8)	0	2 (5.9)	3 (5.8)	
score =3	18 (17.3)	3 (16.7)	9 (26.5)	6 (11.5)	
score =4	12 (18.8)	0	4 (16.6)	8 (24.2)	
New home care ventilator support [b], n (%)	25 (39.1)	1 (14.3)	13 (54.2)	11 (33.3)	0.24
ADL Katz score [c], n (%)					0.37
score =6	35 (54.7)	5 (71.4)	16 (66.6)	14 (42.4)	
score ≤5and ≥3	22 (34.4)	1 (14.3)	6 (25)	15 (45.5)	
score ≤2	7 (10.9)	1 (14.3)	2 (8.3)	4 (12.1)	

Data are presented as mean (percentage)

ADL Activities of daily living, ICU Intensive care unit, mMRC, modified Medical research council

[a] The mMRC scale assesses dyspnea using a 5-point scale based on the sensation of breathing difficulty during daily life activities. Level 0 is the lowest level of perceived dyspnea and level 4 the greatest level of perceived dyspnea.

[b] New home-care ventilatory support was defined as requirement of home-care ventilatory support after hospital discharge in patients with no home-care ventilatory support before ICU admission, and need for CPAP or NIV after hospital discharge in patients having oxygen at home before ICU admission.

[c] The ADL Katz scale assesses functional status as a measurement of the ability to perform activities of daily living (bathing, dressing, toileting, transferring, continence and feeding) independently. Patient score: Yes equals 1 point and No equals 0 point for independence in

each of the six activities. A score of 6 indicates no deficiencies, 5 to 3 indicates mild to moderate deficiencies and 2 to 0 indicates severe deficiencies.
