# Supplementary material for: One year after ICU admission for severe community-acquired pneumonia of bacterial, viral or unidentified etiology. What are the outcomes?
Source: PLoS One. 2020 Dec 14;15(12):e0243762. doi: 10.1371/journal.pone.0243762 (PMC7735561; doi:10.1371/journal.pone.0243762)
Supplement: S1 Fig — SOFA = Sepsis-related organ failure assessment. (PDF) [file pone.0243762.s009.pdf]

S1 Fig. Multivariate analysis of factors associated with one-year mortality of 123 patients analyzed when the variables age and diabetes were forced into the model.

	Hazard-Ratio	Lower limit	Upper limit	p-Value
Bacterial group (vs. others)	2.150	1.021	4.528	0.044
Chronic respiratory failure	1.390	0.691	2.794	0.356
Chronic heart failure	1.616	0.829	3.149	0.159
SOFA	2.657	1.187	5.949	0.018
Immunocompromised	1.981	0.995	3.947	0.052
Age	1.024	0.994	1.055	0.119
Diabetes	0.360	0.146	0.886	0.026

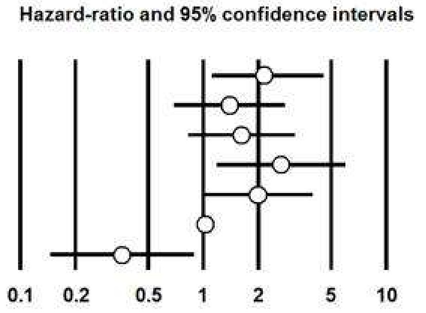

SOFA = Sepsis-related organ failure assessment
