# Supplementary figures and images for: One year after ICU admission for severe community-acquired pneumonia of bacterial, viral or unidentified etiology. What are the outcomes?
Source: PLoS One. 2020 Dec 14;15(12):e0243762. doi: 10.1371/journal.pone.0243762 (PMC7735561; doi:10.1371/journal.pone.0243762)

**S1 Fig: Kaplan-Meier plots of long-term survival after ICU admission in the three groups**

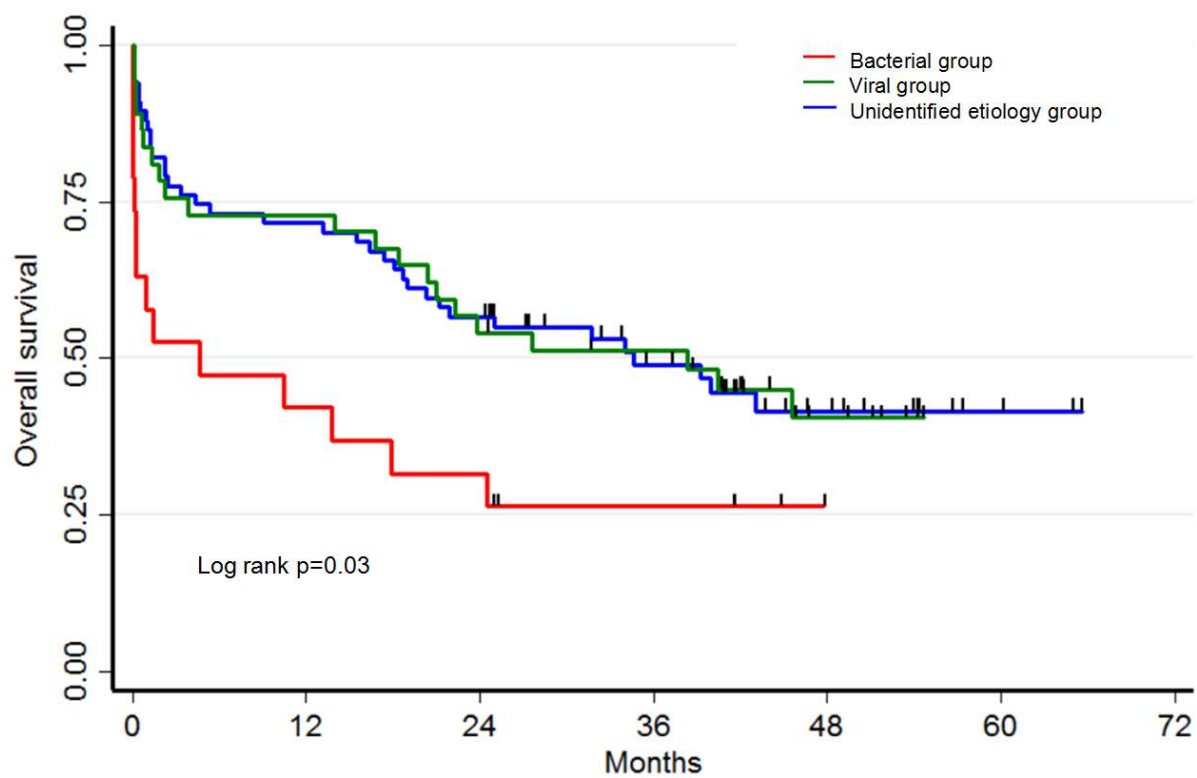

Supplement: S2 Fig — (PDF) [file pone.0243762.s010.pdf]
